# PGPB Consortium Formulation to Increase Fermentable Sugar in *Agave tequilana* Weber var. Blue: A Study in the Field

**DOI:** 10.3390/plants13101371

**Published:** 2024-05-15

**Authors:** Beatriz G. Guardado-Fierros, Diego A. Tuesta-Popolizio, Miguel A. Lorenzo-Santiago, Ramón Rubio-Cortés, Rosa M. Camacho-Ruíz, José J. Castañeda-Nava, Antonia Gutiérrez-Mora, Silvia M. Contreras-Ramos

**Affiliations:** 1 Unidad de Tecnología Ambiental, Centro de Investigación y Asistencia en Tecnología y Diseño del Estado de Jalisco A.C. (CIATEJ), Normalistas No. 800, Colinas de la Normal, Guadalajara 44270, Jalisco, Mexico; beguardado_al@ciatej.edu.mx (B.G.G.-F.); biodgo@gmail.com (D.A.T.-P.); posdoc_mlorenzo@ciatej.edu.mx (M.A.L.-S.); 2 Promoción y Fomento de Agave S. de R.L. de C.V., Ignacio Zaragosa #55 C.P., Ixtlahuacán del Río 45260, Jalisco, Mexico; ramon.rubio@beamsuntory.com; 3 Unidad de Biotecnología Industrial, CIATEJ, Camino Arenero 127, Zapopan 44270, Jalisco, Mexico; rcamacho@ciatej.mx; 4 Unidad de Biotecnología Vegetal, CIATEJ, Camino Arenero 127, Zapopan 44270, Jalisco, Mexico; jcastaneda@ciatej.mx (J.J.C.-N.); agutierrez@ciatej.mx (A.G.-M.)

**Keywords:** agave, inulin, PGPB, tequila beverage, sugars

## Abstract

*Agave tequilana* Weber var. Blue is used as the primary raw material in tequila production due to its fructans (inulin) content. This study evaluates the formulation of a plant-growth-promoting bacteria (PGPB) consortium (*Pseudomonas* sp. and *Shimwellia* sp.) to increase sugars in *A. tequilana* under field conditions. A total of three doses were tested: low (5 L ha^−1^), medium (10 L ha^−1^), and high (15 L ha^−1^), with a cellular density of 1 × 10^8^ CFU mL^−1^ and one control treatment (without application). Total reducing sugars (TRS), inulin, sucrose, glucose, fructose, and plant growth were measured in agave plants aged 4–5 years at 0 (T0), 3 (T3), 6 (T6), and 12 (T12) months. Yield was recorded at T12. The TRS increased by 3%, and inulin by 5.3% in the high-dose treatment compared to the control at T12. Additionally, a low content of sucrose, glucose, and fructose (approximately 1%) was detected. At T12, the weight of agave heads increased by 31.2% in the medium dose and 22.3% in the high dose compared to the control. The high dose provided a higher inulin content. The *A. tequilana* plants were five years old and exhibited growth comparable to the standards for 6–7-year-old plants. This study demonstrates a sustainable strategy for tequila production, optimizing the use of natural resources and enhancing industry performance through increased sugar content and yield.

## 1. Introduction

The Food and Agriculture Organization of the United Nations (FAO) reported in 2021 that 38% of the Earth’s land surface is used for agricultural activities [1]. Currently, worldwide, the countryside is under constant pressure to maintain the ecosystem services it provides, including high yields and food demand. 

The latest report on pesticide consumption indicated an application of about 2.6 million tons worldwide, of which 1.4 million tons were used in America in 2020; around 65% were herbicides, 14% were fungicides and bactericides, and 13% were insecticides [2]. In Mexico, 14% of the territory is used for agricultural production, equivalent to 27.4 million hectares [3]. It is estimated that agricultural lands in Mexico receive pesticide applications ranging from 1 to 2.5 kg ha^−1^, nitrogen fertilizers between 50 to 100 kg ha^−1^, phosphorus (P2O5) at rates from 20 to 35 kg ha^−1^, and potassium at levels of 5 to 15 kg ha^−1^ [4].

So, agriculture faces a critical challenge: intensifying production while using natural resources responsibly. The FAO warns that inappropriate water and land use can threaten food security and environmental sustainability. In this context, strategies must be sought to optimize production without compromising resources [5]. Also, FAO has recommended using a combination of biotechnological tools to support the problems faced by agriculture in the care of soil resources and improve the promotion of agroecological alternatives [1]. 

One of the alternatives is the use of biofertilizers or bioinoculants, which are defined as “a substance which contains living or dormant microorganisms when applied to seed, plant surfaces, or soil, colonizes the rhizosphere or the interior of the plant and promotes growth by increasing the supply or availability of primary nutrients to the host plant” [6,7]. In the last few years, the biofertilizer or bioinoculants market has had a cumulative annual growth rate of 14.08% (from 2016 to 2022) [8]. North America (USA, Canada, and Mexico) dominates the biofertilizer market, followed by Europe, with leading countries such as Germany, the UK, Spain, Italy, Hungary, and France. In the Asia-Pacific region are leading countries such as China, Japan, India, Australia, New Zealand, and the rest of Asia. The biofertilizer global market is estimated to reach USD 3.5 billion by 2025 [6,9,10]. 

Some bacteria used in biofertilizers belong to plant-growth-promoting bacteria (PGPB) because they can colonize plant roots and facilitate plant growth through direct or indirect mechanisms. 

PGPB has different mechanisms of action, such as nitrogen fixation, phosphate solubilization, phytohormone production, protection against pathogens, and disease suppression. It directly enhances plant growth and development, promoting more robust root systems and increased biomass, which make plants more resilient by improving their tolerance to environmental stresses such as drought, salinity, and temperature extremes [11,12,13,14,15]. In the global market, nitrogen-fixing microorganisms represent 80%, followed by the phosphate-solubilizer (14%) [9,10]. Genera such as *Rhizobium* spp., *Azotobacter* spp., and *Azospirillum* spp. are nitrogen-fixing in the global market [6].

The benefits of using PGPB extend beyond plant growth promotion. Several studies have demonstrated that PGPB increases sugar content in various plants, such as tomatoes [16], pears [17], blueberries [18], or strawberries [19], by improving nutrient uptake, photosynthesis, and water use efficiency, increasing fructose, glucose, sucrose accumulation and reducing citric acid metabolism in plants [20]. In addition, PGPB improves growth, yield, antioxidant content, and nutritional quality. These studies demonstrate the potential of utilizing PGPB to enhance fruit sugar content, improve quality, and increase yields, providing valuable insights for sustainable agricultural practices, especially in high-value crops.

In Mexico, *Agave tequilana* Weber, commonly known as the blue agave, is a plant endemic to Mexico and is highly significant in the production of tequila, a renowned Mexican alcoholic beverage with an appellation of origin [21]. Approximately 163 species of the *Agave* genus grow in Mexico, and 123 species are endemic [22]. The production of tequila is indeed restricted to the blue agave variety, ensuring the authenticity and quality of the beverage [23]. In 2022, 143,866 ha were planted with *Agave tequilana* Weber var. Blue [24], and it was estimated that 2288 thousand tons were harvested and used for tequila production in 2023 [25].

The agave plants are characterized by their fructan-rich composition (fructose-based polysaccharides). In *Agave* species (Crassulacean acid metabolism (CAM) plants), fructans are carbohydrate reserves synthesized and stored in the stems. Their primary function is storage before flowering, and they act as osmoprotectants during drought [26]. In tequila production, the fructans from agave are hydrolyzed by cooking, and after the resultant sugar is fermented, the product is distilled to obtain the tequila beverage [27]. So, fructans are the main raw material in the tequila industry; however, they have other industrial applications, such as prebiotic compounds, in the food and cosmetic industry [23,28,29,30,31].

This study aimed to evaluate a PGPB consortium formulation to increase fermentable sugar in *Agave tequilana* Weber var. Blue under field conditions for one year. The plant’s growth was recorded, and yield was measured by harvested head weight. This study is expected to contribute to developing sustainable tequila production strategies, optimizing natural resource use, and the industry’s performance.

## 2. Results and Discussion

### 2.1. Total Reducing Sugars (TRS)

**Figure 1:**
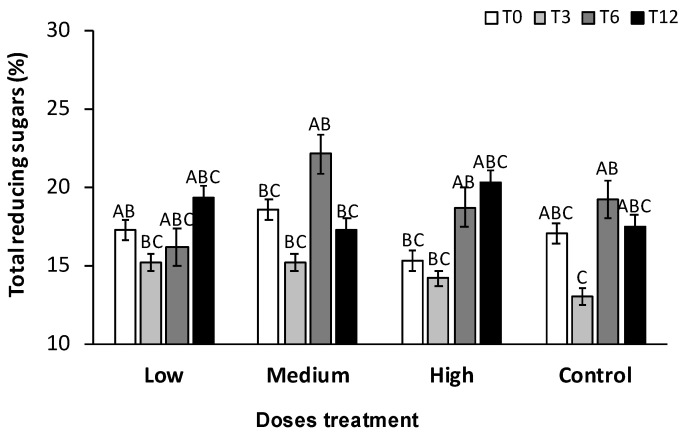
Total reducing sugars percentage in low, medium, and high doses of PGPB consortium and control treatment during the experiment at 0 (T0), 3 (T3), 6 (T6), and 12 (T12) months. Bars indicate standard error. Different capital letters indicate significant differences between treatments and control at the same sampling time for the Dunn test (*p* < 0.05).

An increase in total reducing sugars (TRS) concentration was observed in all treatments evaluated. At T6, a higher TRS content (22.2%) was found in a medium dose of PGPB consortium treatment compared to control plants (19.2%) (*p* >0.05). Nonetheless, at T12, a higher concentration (20.4%) was found in high dose applied, while in control, 17.6% was recorded (Figure 1). An increase of approximately 3% was observed in TRS in high-dose treatment compared to the control at T12. However, no significant differences were found (*p* > 0.05). The average TRS at T0 in agave plants was 17%; a decrease from 1% to 3% in PGPB consortium treatment was observed at T3 (rainy season), and in control, it decreased by 4%. During the field experiment, it was observed that at T3, the soil was flooded, leading to a dilution of TRS, possibly due to water uptake by the plant. Plant control showed no incremental global change from T0 to T12 in TRS content (Figure 1). 

The development of *Agave tequilana* weber var. Blue requires 7 to 10 years, with TRS content fluctuating between 16% and 28% [32]. The age of the agaves in this study was 4–5 years, reaching a TRS content of up to 20.38% (high dose PGPB consortium). The Tequila Regulatory Council (CRT by Spanish singles) reported in its database an average of 21.17% of TRS and an interval from 3.33% to 35.15% in 2017 (last data reported) [25].

Early reports indicated that TRS content ranges from 20% to 30% in *A. tequilana*, with 25% to 30% considered high-quality agave heads [33,34]. Bautista-Justo et al. [35] reported a lower TRS concentration (23.68% to 30%) in winter than in spring (dry season) (27.08% to 32.69%) in *A. tequilana* confirming that the highest TRS concentration occurs during the dry season. 

On the other hand, Arrizon et al. [26] determined the TRS content according to the age of *Agave tequilana* weber var. Blue found that at two years, it was 4.27%, while at four years, it reached 9.04%, and at 6.5 years, it reached 12.8%. Zuñiga et al. [36] reported 26.8% to 29% TRS in *A. tequilana* 6-year-old plants when they applied different fertilization treatments with N, P, K, and micronutrients. In other species of Agave, such as *Agave cocui* (age not reported), TRS reached 34.1% [37], while in *Agave angustifolia* and *Agave karwinskii* Zucc (adult age, between 6 to 8 years) presented 21.16% and 27.29%, respectively [38].

### 2.2. Inulin, Sucrose, Glucose, and Fructose

The primary sugar in the agave head to produce alcohol is inulin because it is the raw material at the beginning of tequila production. Inulin (fructans) is hydrolyzed during cooking or juice extraction, and sugars (fructose–glucose) are produced by yeast during fermentation [27]. 

**Figure 2:**
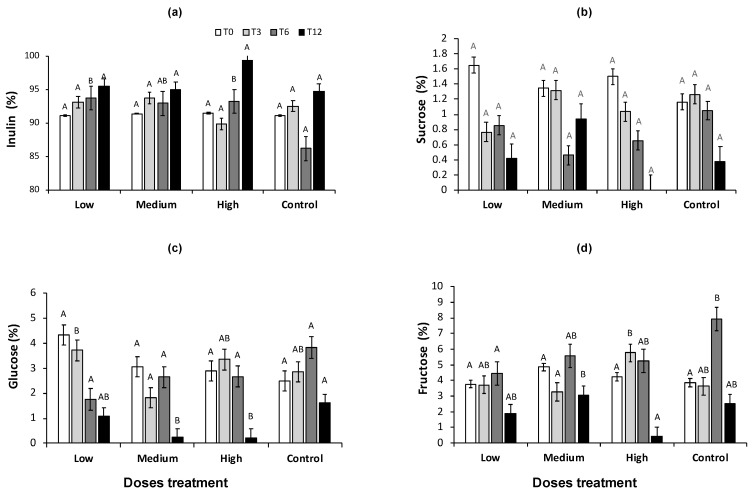
(**a**) Inulin, (**b**) Sucrose, (**c**) Glucose, and (**d**) Fructose percentage in low, medium, and high doses of PGPB consortium and control treatment during the experiment at 0 (T0), 3 (T3), 6 (T6), and 12 (T12) months. Bars indicate standard error. Different capital letters indicate significant differences between treatments and control at the same sampling time for the Dunn test (*p* < 0.05).

The inulin concentration behavior shows an increase in the experiment time from 91% at T0 to 99% at T12, with a significant increase in low and high doses of PGPB consortium compared to control at T6 (Figure 2a) (*p* < 0.05). However, the high-dose treatment of the PGPB consortium showed a higher inulin concentration (99.3%) than other doses evaluated (95%) concerning the control treatment with 94%. A global increase of 7.85% inulin was observed in the high-dose treatment of the PGPB consortium at the end of evaluation T12 concerning T0 (Figure 2a). The control increased by only 3.6% during the same period. This result represents 4.65% more inulin content in the high dose of the PGPB consortium application than in the control (*p* > 0.05).

Early reports by Bautista-Justo et al. [35] indicated that *A. tequilana* had the highest inulin content, from 20% to 24%. Also, Arrizon et al. [26] reported that the percentage of inulin increases according to the age of the plant, so plants 64.98% at two years old, 94.32% at four years old, and set up 97.63% by the age of 6.5 years. These concentrations are lower than those obtained in the present study. However, it is important to highlight that the current study’s concentrations surpass those Arrizon et al. reported.

Inulin or fructans are stored in the agave during the vegetative development of a plant to provide energy for plant growth. So, the primary function is storage before flowering, and it also acts as an osmoprotectant in drought conditions [26,39]. The *A. tequilana* fructan content increased in 4–5-year-old plants and remained constant for up to 7-year-old plants [40]. After that, agave plants reach the physiological reproductive stages, such as shoots and inflorescence (seeds), where their carbohydrate content changes [40].

The benefits to plants when inoculated with PGPB or other beneficial microorganisms (fungi) have been well documented [41,42]; however, in agave plants (CAM), little information is available. The PGPB has been reported by an increase in the sugar content in different fruits of crops, and few reports exist of an increase in inulin or sugar in agave. Torres-Ruiz et al. [43] reported a decrease in inulin content in *A. tequilana* (60 days old) with inoculation of bacterial endophytes with PGPB capacities (*Pseudomonas mosselii*, *Acinetobacter calcoaceticus*, and *Rhizobium daejeonense*) isolated from *A. tequilana* leaves. *Azospirillum brasilense* increased the sugar content in *Agave angustifolia* by 4.85% and 4.96%, compared to the control [44].

The sucrose content in agave plants decreases over time. At the beginning of the experiment, the sucrose content was 1.4% on average (Figure 2b). No significant differences were observed in this sugar between all treatments and control. However, no sucrose was observed in T12 in the high-dose treatment, while 0.38% was detected in the control. In treatments low and medium, 0.42% and 0.94% of sucrose were recorded, respectively (*p* > 0.05) (Figure 2b).

The glucose content decreased over time in all treatments where the PGPB consortium was applied, while in the control, it increased and only at T12 decreased. Glucose content was significantly higher in the control with 1.62% at T12 than in the treatments with low (1.074%), medium (0.236%), and high (0.228%) doses (Figure 2c). In addition, at the beginning of the experiment, the average fructose concentration was 1.18%, and no significant change was observed at T3 or T6 with the different doses of the PGPB consortium. However, at T6, higher fructose content was observed in control agave plants (7.9%) than in treatments (*p* > 0.05). At T12, all treatments decreased the fructose concentration with a lower content in high-dose treatments (0.42%) than control with 2.53% (Figure 2d).

Their carbohydrate content changes when agave plants reach the reproductive stages [40]. The active hydrolysis of fructans by fructane hydrolase results in an increase in fructose and glucose content biosynthesized via CAM, so fructans are hydrolyzed by vacuolar invertase, generating glucose and fructose [43], which the plants use for flowering.

The glucose and fructose concentrations decrease with agave age. Some reports indicated glucose contents as 13.53% at two years old, 0.49% at four years old, and 0.58% at 6.5 years old. At the same time, fructose was reported to be 15.12%, 0.91%, and 0.79%, respectively [26]. Michel-Cuello [45] reported 0.41% glucose and 0.22% fructose content in *A. tequilana,* 3.5 years old. 

In contrast, Zuñiga et al. [36] observed an increase in fructose concentration during the agave head growth stage with a concentration of 24.68%, representing 88% of total monosaccharides. Glucose concentration decreased to 3.53% at the end of the six years. The glucose and fructose concentrations in agave plants from 2 to 7 years have been reported to range from 1.41% to 0.42% for glucose and from 2.08% to 1.17% for fructose, indicating a linear decrease in the concentrations of both monosaccharides [46].

### 2.3. Yield of Agave Head

**Figure 3:**
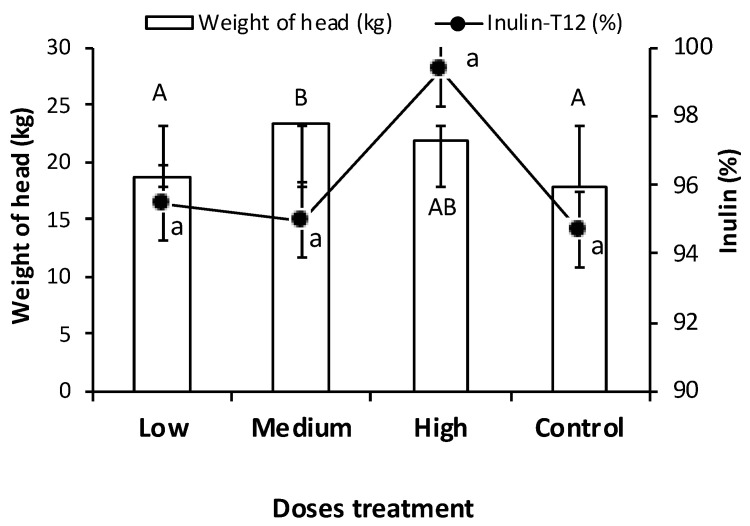
Weight of agave head (kg) and Inulin content (%) at twelve months (T12), in low, medium, and high doses of PGPB consortium and control treatment during the experiment at 12 (T12) months. Bars indicate standard error. Capital letters indicate the comparison between weight treatments, and lowercase letters indicate the comparison for inulin. Different letters indicate significant differences for the Dunn test (*p* < 0.05).

The weight head estimation is of the agave yield. The average weight of the agave head significantly increased with the PGPB consortium (medium dose) application compared to control plants. The higher average weight was found in medium-dose (23.5 kg), followed by high-dose (18.6 kg), while the control presented a weight of 17.9 kg (Figure 3). These results represent an increase of 5.6 kg in the medium dose and 4 kg in the high dose compared to the control. In the experimental field, the plant density was 3500 per ha, so the yield in head agave weight is equivalent to 19.6 tons ha^−1^ and 14 tons ha^−1^, respectively. Furthermore, the treatment with high-dose of the PGPB consortium increased the inulin concentration to T12 (99%). The weight of agave heads increased by 31.2% in the medium dose of the PGPB consortium after 12 months and 22.3% in the high dose compared to the weight of the plant control.

The reported weight by plant’s different ages is as follows: 2 years, 4.51 kg; 4 years, 32.6 kg; and 6.5 years, and 90.9 kg [26]. In 6-year-old plants used as an absolute control, a weight of 14.1 kg was reported, while plants with fertigation had an average weight of 66.1 kg [36]. Plants between 6 and 8 years old have an average weight ranging from 30 to 60 kg per agave head in the Jalisco, Mexico region [47]. These results show that plants exhibit variation in weight due to factors such as age and management.

In addition, it is known that the promoting growth of plants is dose-dependent within a specific range of cell densities [48,49]. The PGPB could have an optimum cell density concentration for its full function, with variations between plants. An excess cell density may cause the loss of symbiotic balance with the host plant, whereas low affinity with the host plant results in a low density of effective cells. The maximum promoting effects on the plant could occur with a specific concentration for each strain, and lower or higher cellular concentrations could result in plant growth reduction, as was demonstrated by Suckstorff and Berg [48] and Natsagdorj et al. [49]. One reason for the dose-dependent effect has been postulated by the complex regulation of secondary metabolites such as N-acyl-LL-homoserinelactones (AHLs), allowing bacteria to sense their population density [48].

Our results showed that the PGPB consortium at a high dose (15 L ha^−1^ with 1 × 10^8^ CFU mL^−1^) had lower weight than the medium dose (10 L ha^−1^ with 1 × 10^8^ CFU mL^−1^) at T12, suggesting that possibly the optimum dose (effective cellular density) could be from 10–15 L ha^−1^ when the interest is increasing agave biomass. However, if the interest is increasing inulin content in the agave head, the high dose of the PGPB consortium could be more effective. At this dose (high), a minimum content of other sugar was detected, which suggested that the responsible enzymes of inulin (fructans) hydrolysis were reduced or inhibited. More studies should be conducted to demonstrate this effect.

### 2.4. Growth Agave

**Figure 4:**
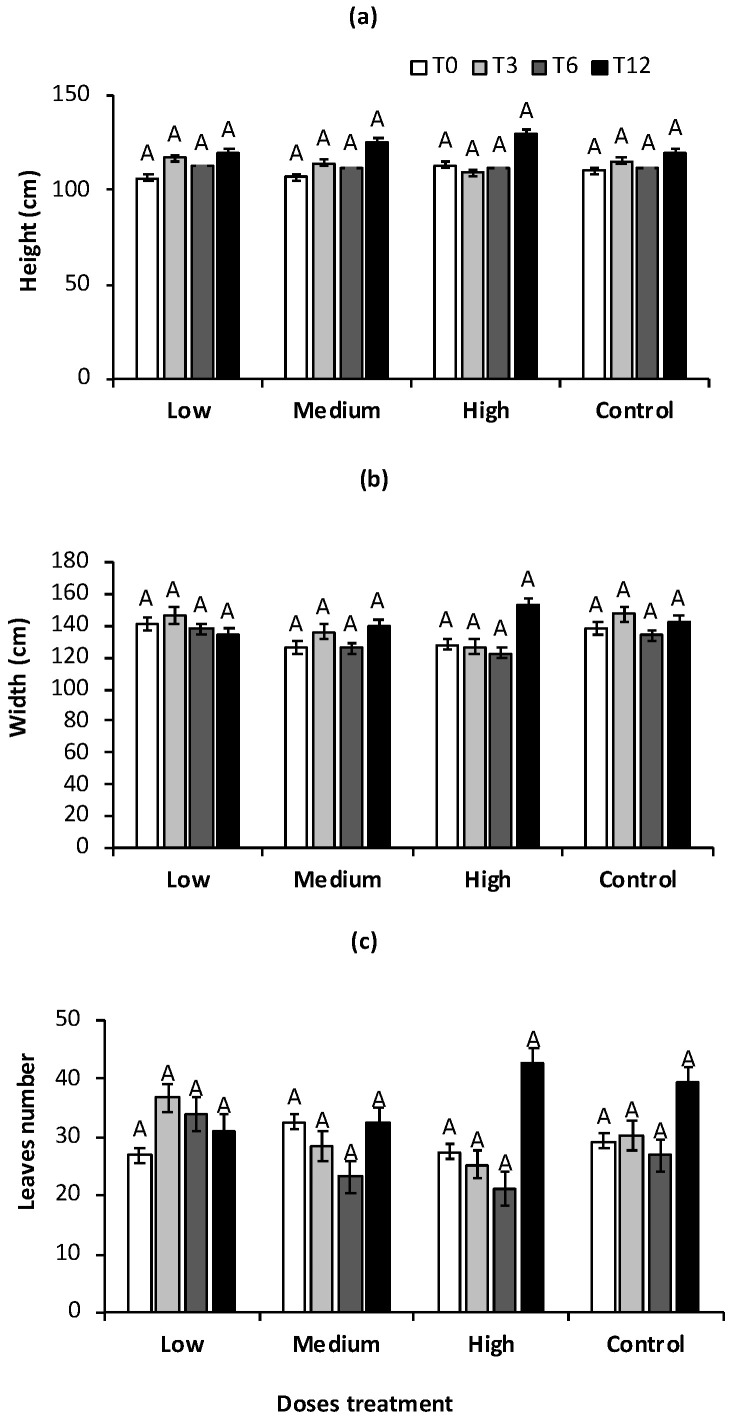
(**a**) Height (cm) of plant, (**b**) Canopy width, and (**c**) Leaves number in low, medium, and high doses of PGPB consortium and control treatment during the experiment at 0 (T0), 3 (T3), 6 (T6), and 12 (T12) months. Bars indicate standard error. Capital letters indicate the comparison between treatments at the same sampling time. Different letters indicate significant differences for the Dunn test (*p* < 0.05).

*A. tequilana* of four years at the beginning of the experiment presented a height of 108 cm; no significant changes were observed at T3 and T6 in treatments with PGPB consortium and control plants. However, an increase in the height of agave plants was recorded at T12 in medium and high-dose treatment. Plants reached 125 cm and 129 cm, respectively, and control plants reached only 119 cm. No statistically significant differences were observed in the plant growth between the different doses of the PGPB consortium tested compared to the control (Figure 4a).

There was less variability in the canopy width of the plants in high doses of the PGPB consortium from T0 to T6; however, an increase in the canopy was recorded (153 cm) without significant differences in control plants (141 cm) at T12 (Figure 4b). *Agave tequilana* plants five years old with the medium dose treatment showed a growth close to the standards of 6–7 years old plants reported. The number of leaves was variable in all treatments and control plants. No significant differences were observed in this parameter between treatments tested. However, a higher number of leaves was detected in agave plants from high doses of PGPB consortium (42 leaves), while in control, it reached 39 leaves (Figure 4c). It is worth mentioning that this measurement may not be precise because leaves are pruned during growth as part of agroecological management.

Some authors have reported the height of *A. tequilana* into intervals 135–210 cm and a canopy width of 142–270 cm, while *A. agustifolia* height was reported to be 161–236 cm and canopy width 236–332 cm [50].

The number of leaves on a four- or five-year-old agave plant can vary by agave species, environmental conditions, and agroecological management [51,52]. There are few reports where the total number of leaves was recorded. *A. tequilana* plants are already two years old and set up a maximum of 15 leaves [52]. Martínez-Ramírez et al. [51] reported that *A. potatorum* plants between 2 and 5 years old had a linear growth phase, while the number of unfolded leaves is in the deceleration phase of growth before flowering. These authors recorded agave plants with 54 leaves without nitrogen fertigation and 58 leaves in agave receiving 100 kg N ha^−1^ in 5-year-old agave plants. A higher number of leaves means more organs producing photoassimilates such as sugars in the plant.

## 3. Materials and Methods

### 3.1. Experimental Site and Soil Characteristics

The experimental site was in Ixtlahuacán del Río, Jalisco, Mexico (21°0′49″ N y 10,312′11″ O). Its average altitude is 1709 m above sea level. It is characterized by a sub-humid and semi-warm climate with a mean annual temperature of 19.6 °C and average annual precipitation of 916 mm, mainly from June through September. The soil in this area was phaeozem cultivated with *Agave tequilana* Weber var. Blue without receiving irrigation (http://www.inegi.gob.mx, accessed on 28 May 2021).

The clay loam soil [53] (USDA modified soil texture triangle) had pH in water 6.2 ± 0.4 [1,54], 0.4 ± 0.1 dS m^−1^ electrical conductivity, 8.0 ± 4.1 meq/100 g cation exchange capacity (CIC) contained 478 ± 16.7 g clay kg^−1^, 302 ± 8.1 g silt kg^−1^ and 219 ± 8.6 g sand kg^−1^, 1.5 ± 0.4% organic matter, 15.0 ± 2.0 mg kg^−1^ total phosphorous (TP) [54,55].

### 3.2. PGPB Consortium

The plant-growth-promoting bacteria (PGPB) consortium (CIATEJ-MX1) was formed by genera of *Pseudomonas* sp. and *Shimwellia* sp. and deposited in ATCC (American Type Culture Collection) with record PTA-122530 and with access number in GenBank (National Center for Biotechnology Information, NCBI) OM966648-OM9666653. They were included in MX/a/2015/015919 patent grant and deposited in microorganism bank Centro Nacional de Recursos Genéticos (CNRG-INIFAP) with record card CM-CNRG-TB204-TB208. Bacteria were isolated from agricultural soil from Zapotlanejo, Jalisco, Mexico (20°38′49.02″ N and 103°2′55.35″ W at 1546 m a.s.l.) and from a fermentative process from organic wastes of two months (combination of humus’s earthworm, whey, molasse, and cow manure). They were previously selected for their plant growth promotion capacity (Table 1). The bacterial consortium can be found in the products COFACTOR^®^ and FXBIO^®^ commercially available at an average 1.0 × 10^8^ CFU mL^−1^ density in the product (https://www.buscador.portaltecnoagricola.com/vademecum/mex/producto/37317/COFACTOR?empresa=POLAQUIMIA and http://ecosystembeta.com/files/, accessed on 28 May 2021). Where the consortium composition is 1.0 × 10^8^ CFU mL^−1^ *Pseudomonas stutzeri*, 1.1 × 10^8^ CFU mL^−1^ *Pseudomonas fluorescens*, 1.1 × 10^8^ CFU mL^−1^ *Pseudomonas koreensis*, 1.2 × 10^8^ CFU mL^−1^ *Shimwellia blattae*. Table 1 describes these strains.

### 3.3. Experimental Setup

**Figure 5:**
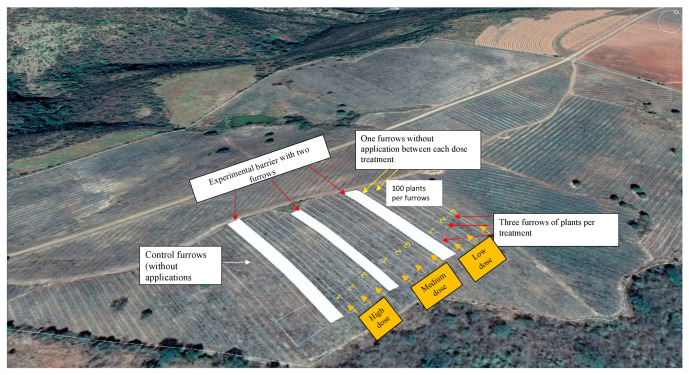
Experimental site with distribution of experimental treatments and furrows. Image obtained with Google Earth software^®^ (7.3.6 Version).

**Figure 6:**
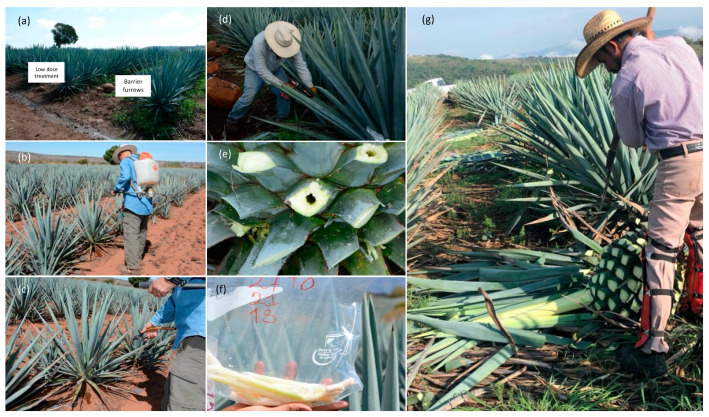
(**a**) Furrows with a dose of PGPB consortium treatment and barrier furrows; (**b**,**c**) PGPB consortium application near the root from the head plant with a backpack sprayer; (**d**) sampling agave head tissue perforated with a core dill bit subject to an electric drill; (**e**) agave plants with perforation after sampling (**f**) cylindrical tissues obtained for analysis. (**g**) Harvest the agave to obtain the head for the final yield.

The experiment was established in a field of agave cultivated using 1200 agave plants four years old without offshoots. The field was divided into four sections to establish three treatments with three different doses of PGPB consortium application indicated as low (5 L ha^−1^), medium (10 L ha^−1^), and high (15 L ha^−1^) and one treatment as control (without application). Each treatment had three lines of furrows, each with 100 Agave plants (n = 300) and separated by one furrow plant without treatment. Also, each dose treatment was separated by two furrows of plants without treatment as a barrier for treated plants (Figure 5 and Figure 6a). Plants received two doses of PGPB consortium at zero time (T0) (June 2021) and six months (T6), which were applied to soil near the root from the head plant with a backpack sprayer (Figure 6b,c). Plants were numbered 1 to 100, leaving the five first plants in the furrows without applications (as a barrier) to the path.

Sortea2 randomly selected ten plants (www.sortea2.com, accessed on 15 June 2021), a digital application that generates random numbers at intervals given by the users, for example, from 1 to 100. It had similar functions as https://www.random.org/ (accessed on 8 September 2021) at 0, 3, 6, and 12 months (T0, T3, T6, and T12). Leaves were cut, agave heads were perforated with a core dill bit subject to an electric drill (Milwaukee^®^ M18™ Fuel, Brookfield, WI, USA), and tissue of the core head plant was obtained (Figure 6a–g). Sub-samples of tissue from three agave plants were mixed for total reducing sugar, and fructans (were measured as inulin), saccharose, glucose, and fructose analysis, described below.

In addition, plant height (topsoil to central leaf), plant width (diameter of leaves extension), and leaf number from all plants were measured and recorded to follow the growth of agave plants in the different treatments. All plants were harvested at 12 months (T12), and the weight of agave heads was recorded in all treatment plants (Figure 6e).

### 3.4. Total Reducing Sugars

A ten g mass of agave tissue was ground to determine total reducing sugars (TRS) with 300 mL of distilled water at 80 °C for 1 min, and the extract was filtered. The Agave juices were diluted 1:1 (*v*/*v*) with distilled water, 5 mL of HCl (50%) was added to 100 mL of the sample, and the samples were incubated for 5 min at 70 °C. The samples were neutralized with NaOH equimolar. Subsequently, the DNS reactive (dinitrosalicylic acid) was prepared. Solution A: NaOH (1.6 g) and KNaC_4_H_4_O_6_ (30 g) diluted in 50 mL of deionized water. Solution B: DNS (1 g) diluted in 30 mL of deionized water. The solution A and B were mixed and filled up to 100 mL. In vials of 2 mL, 0.5 mL of hydrolyzed sample and 0.5 mL of mixed DNS (A + B solution) were collocated at 100 °C for 5 min. The D-fructose curve ranges from 0 to 1000 mg L^─1^. The samples were analyzed using the DR 5000™ UV-Vis Spectrophotometer (Hach, Mississauga, ON, Canada), with measurements taken at 540 nm [59].

### 3.5. Inulin, Glucose, Sucrose, and Fructose

Different sugars (glucose, fructose, sucrose, and inulin) were determined by methods previously reported by Aragón-León et al. [28] on an HPLC system (Agilent, Alpharetta, GA, USA) coupled with a refractive index detector. The chromatographic column used for ion exchange was Aminex HPX-87C (7.8 mm × 300 mm, Bio-Rad, Hercules, CA, USA). HPLC-grade water with a flow rate of 0.55 mL min^−1^ was utilized as the mobile phase. The injected sample volume was 20 μL, and the column temperature was 68 °C. Before analysis, the samples were diluted and filtered using polypropylene-membrane filters (0.45 μm, Agilent, Alpharetta, GA, USA). Sugar quantification was conducted based on the calibration curves. The compounds were quantified in triplicate samples by preparing a calibration curve of a mixture of glucose, fructose, sucrose (Sigma-Aldrich^®^, Saint Louis, MO, USA), and from chicory inulin (Raftiline HP^®^, BENEO Inc., Parsippany-Troy Hills, NJ, USA), with each standard compound at 1, 2, 4, 6, 8, 10, 1,6, and 20 g L^−1^ [28,60].

### 3.6. Statistical Analysis

Total reducing sugar, inulin, saccharose, glucose, and fructose percentage data were submitted to Bliss transformation (X* = arcosen √(X/100)), where X* is the transformed data, and X is the original data in percentage. After that, all data were submitted to normality and Kruskal–Wallis test and compared with Dunn’s multiple range tests at *p* ≤ 0.05 to determine significant differences between treatments using the XLSTAT (2021) software. The abstract figure in this document was produced using Biorender software (www.biorender.com, accessed on 28 June 2022).

## 4. Conclusions

Applying the PGPB consortium to *A. tequilana* increased the total reducing sugars (TRS), inulin (fructans), and weight of agave heads in the medium- and high-dose treatment compared to the control at 12 months. Both treatments exhibited potential benefits in enhancing the agave head weight. Notably, the high-dose treatment demonstrated a higher inulin content (99%) in the agave head. *Agave tequilana* plants treated with the medium-dose treatment at five years showed a growth close to the standards observed in 6–7-year-old plants reported. This study contributes to developing sustainable strategies for tequila production, optimizing natural resource utilization, and enhancing industry performance with a high sugar content and yield. Future experiments could further optimize the PGPB consortium’s effect on agave plants and other value crops.

## Figures and Tables

**Table 1:** Consortium composition, strain identification, access number GenBank and Indole-3acetic acid (IAA) with or without tryptophan, phosphorous solubilization, siderophores percentage production and morphology by Gram staining.

Strain	Strain ID	Access Number GenBank	IAA with Tryptophan (mg L^−1^) [56]	IAA without Tryptophan (mg L^−1^) [56]	Phosphorus Solubilization (mg L^−1^) [57]	Siderophores (%) [58]	Gram Staining
*Pseudomonas stutzeri*	strain 9-2	OM966649	24	10.4	15	90%	Bacilli (-)
*Pseudomonas fluorescens*	strain 19-1	OM9666651	10.1	8.1	10	50%	Bacilli (-)
*Pseudomonas koreensis*	strain 9-3	OM966650	32.0	27.4	18	10%	Bacilli (-)
*Shimwellia blattae*	strain 25-2	OM966653	3.6	4.0	8.8	50%	Bacilli (-)

## Data Availability

Data will be available at the request of any reader.
